# HIPK2-T566 autophosphorylation diversely contributes to UV- and doxorubicin-induced HIPK2 activation

**DOI:** 10.18632/oncotarget.14421

**Published:** 2017-01-02

**Authors:** Alessandra Verdina, Giuliana Di Rocco, Ilaria Virdia, Laura Monteonofrio, Veronica Gatti, Eleonora Policicchio, Alessandro Bruselles, Marco Tartaglia, Silvia Soddu

**Affiliations:** ^1^ Unit of Cellular Networks and Molecular Therapeutic Targets, Department of Research, Advanced Diagnostics, and Technological Innovation, Regina Elena National Cancer Institute - IRCCS, Rome, Italy; ^2^ Department of Hematology, Oncology and Molecular Medicine, Istituto Superiore di Sanità, Rome, Italy; ^3^ Genetics and Rare Diseases Research Division, Ospedale Pediatrico Bambino Gesù – IRCCS, Rome, Italy; ^4^ Present address: Istituto di Biologia Cellulare e Neurobiologia, CNR, Monterotondo Scalo, Rome, Italy

**Keywords:** HIPK2, phosphorylation, DNA-damage response, cancer stem cells

## Abstract

HIPK2 is a Y-regulated S/T kinase involved in various cellular processes, including cell-fate decision during development and DNA damage response. *Cis*-autophosphorylation in the activation-loop and *trans*-autophosphorylation at several S/T sites along the protein are required for HIPK2 activation, subcellular localization, and subsequent posttranslational modifications. The specific function of a few of these autophosphorylations has been recently clarified; however, most of the sites found phosphorylated by mass spectrometry in human and/or mouse HIPK2 are still uncharacterized. In the process of studying HIPK2 in human colorectal cancers, we identified a mutation (T566P) in a site we previously found autophosphorylated in mouse Hipk2. Biochemical and functional characterization of this site showed that compared to wild type (wt) HIPK2, HIPK2-T566P maintains nuclear-speckle localization and has only a mild reduction in kinase and growth arresting activities upon overexpression. Next, we assessed cell response following UV-irradiation or treatment with doxorubicin, two well-known HIPK2 activators, by evaluating cell number and viability, p53-Ser46 phosphorylation, p21 induction, and caspase cleavage. Interestingly, cells expressing HIPK2-T566P mutant did not respond to UV-irradiation, while behaved similarly to wt HIPK2 upon doxorubicin-treatment. Evaluation of HIPK2-T566 phosphorylation status by a T566-phospho-specific antibody showed constitutive phosphorylation in unstressed cells, which was maintained after doxorubicin-treatment but inhibited by UV-irradiation. Taken together, these data show that HIPK2-T566 phosphorylation contributes to UV-induced HIPK2 activity but it is dispensable for doxorubicin response.

## INTRODUCTION

HIPK2 (Homeodomain-Interacting Protein Kinase 2) is an evolutionarily conserved tyrosine-regulated serine/threonine kinase. HIPK2 phosphorylates a large body of proteins belonging to different signaling pathways involved in the control of development, cell response to DNA damage and hypoxia, differentiation, and cytokinesis [reviewed in 1-4].

In physiological conditions, HIPK2 localizes mainly in nuclear speckles, including PML-bodies, and only a small fraction is found in the nucleoplasm or cytosol [5]. In unstressed cells, HIPK2 is constantly degraded by the ubiquitin-proteasome system [6]. In stressing conditions, such as in response to UV, ionizing radiation, and chemotherapeutic drugs, HIPK2 stability is regulated by different ubiquitin E3 ligases [6–10] and induces cell cycle arrest or apoptosis through p53-dependent and -independent mechanisms [11–15]. In addition to ubiquitylation, HIPK2 is regulated by different posttranslational modifications (PTMs), caspase cleavage [16], and by the interaction with scaffold proteins [9, 17]. Of relevance, HIPK2 PTMs have been shown to occur in a hierarchical fashion, with phosphorylation being required for sumoylation, and the latter controlling acetylation [4, 18].

Thus far, different RefSeqs have been used for HIPK2 aminoacid numbering. Here, we will refer to the current NCBI RefSeq (*i.e*., NP_073577.3 and NP_034563.2 for human and mouse HIPK2, respectively) and, when required, add in brackets the aminoacid number used in the relative references. Both human and mouse HIPK2 have been shown to become catalytically active by their *cis*-autophosphorylation of the activation loop at Y361 (Y354) and S364 (S357) [19–21]. Of relevance, the *cis*-autophosphorylation also controls the subcellular localization of HIPK2 and its substrate affinity [19–21]. Subsequent HIPK2 phosphorylations take place at multiple sites (>40 sites pooling current mouse and human data) distributed throughout the different HIPK2 domains both by *trans*-autophosphorylation at S/T sites and phosphorylation by other, mostly unknown kinases, in S/T and Y sites (Table 1). The functional consequences of a few of these phosphorylations are becoming clear. For example, in DNA damage response (DDR), HIPK2 activation and accumulation is induced by its autophosphorylation at T880/S882 that creates a binding signal for the phospho-specific isomerase Pin1 [22], or by c-Abl-mediated phosphorylation at Y367 (Y360) [23]. An inhibitory phosphorylation of HIPK2 by AMPKα2 at T119 (T112), S121 (S114), and T1114 (T1107) has been shown to release WIP1, a homeostatic regulator of DDR, from the HIPK2-mediated phosphorylation and degradation [24]. More recently, HIPK2 phosphorylation at S359/T360 in the activation loop has been causally linked to ER stress-mediated neurodegeneration in amyotrophic lateral sclerosis [25]. In contrast, substitutions of the putative S/T phosphorylation sites at position S118 (S111), S121 (S114), S441 (S434), T450 (T443), and T517 (T510) with non-phosphorylatable alanine did not reduce HIPK2 kinase activity in *in vitro* assays [19].

**Table 1:** Human and mouse HIPK2 phosphorylation sites

Site*	Human HIPK2	MouseHipk2	Kinase	References
S16		x	Auto(P)	[20]
Y44	x		Src-overexpressing cells	[49]
S118	x	x	Auto(P)	[19, 20]
T119	x		AMPKα2	[24]
S121	x		AMPKα2	[24]
S135		x	n.d.	[20]
T141		x	n.d.	[20]
T252		x	n.d.	[20]
Y258	x		Src-overexpressing cells	[49]
Y264	x		Src-overexpressing cells	[49]
T273		x	Auto(P)	[20]
S359	x		ER-stress in ALS	[25, 50, 51]
T360	x	x	ER-stress in ALS	[23, 25, 52, 53]
Y361	x	x	cis-Auto(P)	[19, 20, 54, 55]
S364	x	x	cis-Auto(P)	[19, 50, 51, 56, 57, 58, 59]
Y367	x		c-Abl	[23]
Y423	x		Src-overexpressing cells	[49]
S441	x	x	Auto(P)	[19, 20]
Y443	x		Src-overexpressing cells	[49]
T482		x	n.d.	[20]
T517	x	x	n.d.	[19, 20]
Y558	x		Src-overexpressing cells	[49]
T566	x**	x	Auto(P)	[20]
S634		x	n.d.	[20]
S668	x	x	Auto(P)	[19, 20, 22]
T687		x	n.d.	[20]
S815		x	n.d.	[20]
S826	x			[19]
S827	x	x	Auto(P)	[19, 20, 22, 59, 60, 61, 62]
T838	x		Auto(P)	[19, 22, 62]
S848	x	x	Auto(P)	[19, 20, 22]
T880	x		Auto(P)	[22]
S882	x		Auto(P)	[22]
S924	x		Auto(P)	[22]
T933	x		Auto(P)	[19]
S934	x	x	Auto(P)	[20, 22]
S955	x		Auto(P)	[19]
S977	x		Auto(P)	[19]
S991		x	Auto(P)	[20]
S993		x	Auto(P)	[20]
S1014	x		Auto(P)	[19]
S1042		x	Auto(P)	[20]
T1114	x		AMPKα2	[24]
Y1136	x		Src-overexpressing cells	[38]
S1153		x	Auto(P)	[20]
S1186		x	Auto(P)	[20]
Y1197	x		Src-overexpressing cells	[38]

* The current NCBI RefSeq, NP_073577.3 for human HIPK2 and NP_034563.2 for mouse Hipk2 proteins have been used. **This manuscript.

HIPK2 dysregulation has been shown to contribute to proliferative diseases, such as cancer and tissue fibrosis [2, 26–28]. Based on our current knowledge, three possible mechanisms explaining the functional inactivation of HIPK2 have been proposed: the “localization model”, the “optimum model”, and the “PTM model” which foresee, respectively, tight regulations of HIPK2 subcellular localization, protein levels, and PTMs [18]. Mutations in the *HIPK2* gene are sporadic in human cancers (Catalogue of somatic mutations in cancer - COSMIC http://cancer.sanger.ac.uk/cosmic) and their contribution to HIPK2 dysregulation has only been rarely verified [29]. By whole-exome sequencing of patient-derived colorectal cancer stem cells (C-CSCs), we identified a mutation (T566P) in one of the sites we previously found autophosphorylated in mouse Hipk2 (T599 in ref. 20) (Table 1) [20]. Here, we investigated the contribution of this site in the HIPK2 activity. We observed an impairment of UV-induced HIPK2 reaction while the HIPK2-mediated response to doxorubicin was not affected, indicating the existence of a DDR-specific phospho-mediated regulation of this kinase.

## RESULTS AND DISCUSSION

In the frame of an effort directed to profile and functionally characterize the cancer driving events affecting a panel of C-CSCs obtained by selective culture from colorectal tumor specimens [30], whole-exome sequencing was carried out using genomic DNA from 24 C-CSC lines and patient-matched non-tumor tissues. Statistics and data output is reported in Supplementary Table 1. Among the multiple hits identified, two different somatically acquired missense changes, c.1694A>C (p.Lys565Thr, K565T hereafter) (line CTSC85) and c.1696A>C (p.Thr566Pro, T566P hereafter) (line CTSC47) (Supplementary Figure 1A), affecting *HIPK2* were identified. Both residues were almost invariably conserved among vertebrates and were located in a highly conserved amino acid stretch (Supplementary Figure 1B), strongly suggesting a possible functional impact of both changes. Consistently, both substitutions were predicted to be damaging by Combined Annotation Dependent Depletion prediction tool [31]. Of note, H565 had previously shown to be an ubiquitylation site and its substitution had been shown to reduce Siah1-mediated degradation in the absence of any appreciable effect on the catalytic activity of the kinase [6]. On the other hand, T566 was previously recognized as one of the sites undergoing reversible autophosphorylation in mouse Hipk2, which suggested a possible role of T566 phosphorylation in the control/modulation of HIPK2 function, and a direct impact of the T566P change on perturbing HIPK2 activity by affecting phosphorylation at T566.

To evaluate whether the T566P amino acid substitution is relevant for HIPK2 localization and activity, we first introduced the T566P mutation in an EGFP-tagged wt HIPK2-expressing vector. Mutation of the nearby K565 to T, found in C-CSC from a different patient (Supplementary Figure 1A), was engineered as control. Transfection of these three vectors (WT, T566P, and K565T) was performed by electroporation in wt p53 carrying U2OS cells. Mock transfected cells (MOCK) and cells transfected with the EGFP empty vector (GFP) were used as control.

**Figure 1:**
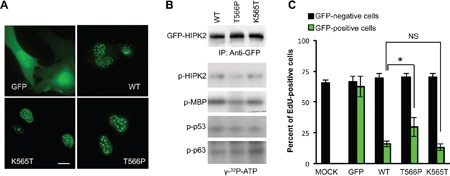
Effect of the T566P mutation on cellular localization, HIPK2 kinase activity, and cell proliferation **A**. U2OS cells were transfected with expression vectors for the indicated GFP-tagged HIPK2 derivative. Protein localization was evaluated by direct GFP fluorescence under UV light. U2OS cells transfected with GFP empty vector (GFP) were used as control. Scale bars 10 μm. **B**. GFP-tagged proteins kinase activity was analyzed by *in vitro* kinase assay by incubation with the recombinant proteins MBP, p53, and p63, in the presence of [γ-^32^P]-ATP. Kinase reaction products were resolved by SDS-PAGE and analyzed by autoradiography. Data from one representative experiment out of three is reported. **C**. U2OS cells were transfected as in (A). EdU-positive cells were detected by fluorescence and counted 24 hrs after transfection. Transfected GFP-positive cells and untransfected GFP-negative cells belong to the same dishes. Data represent the mean ± Standard Error (SE) of at least three different experiments.* P<0.05; NS P>0.05 by Student t test.

HIPK2 mainly localizes into nuclear speckles and specific PTMs, such as *cis*-autophosphorylation, sumoylation, and acetylation can regulate the localization of the kinase. Thus, we first evaluated the localization of our GFP-HIPK2 derivatives. As shown in Figure 1A, T566P mutant has a nuclear-speckled localization comparable to that of wt HIPK2 and HIPK2-K565T mutant, whereas the GFP control showed a diffuse nuclear/cytoplasmic localization, as expected.

Next, we evaluated the kinase activity of the three GFP-HIPK2 derivatives on the kinase its-self, a non-specific substrate, the myelin basic protein (MBP), and two specific substrates, p53 and p63, as previously described [20]. GFP-HIPK2 derivatives overexpressed in U2OS cells were immunopurified and comparable protein amounts employed for *in vitro* kinase assays. Compared to wt HIPK2 and the ubiquitylation mutant, HIPK2-T566P mutant showed a very mild reduction of kinase activity on its-self and on MBP, while no significant difference was observed in the levels of p53 and p63 phosphorylation (Figure 1B).

HIPK2-mediated cell cycle arrest and apoptosis requires its kinase activity [11–13]. To verify whether the mild and partial reduction of the HIPK2-T566P kinase activity might be relevant for its function, we compared the survival and proliferation-suppressing activities of the three GFP-HIPK2 derivatives by EdU (5-ethynyl-2′-deoxyuridine) incorporation upon expression in U2OS cells. A comparable, early and mild induction of cell death was observed among the cell populations transduced with the three EGFP-HIPK2 derivatives (4±0.5%, 6±0.6%, and 9±0.8% for WT, T566P, and K565T, respectively). Single-cell analyses for EdU incorporation and staining of GFP-positive, transfected cells, and GFP-negative, untransfected cells in the same dish showed a significant inhibition of EdU positivity in the wt HIPK2 and HIPK2-K565T expressing cells compared to relative GFP-negative cells. In contrast, the HIPK2-T566P mutant was consistently less efficient in suppressing EdU incorporation (Figure 1C). Overall, these data documented that mutation of the autophosphorylation HIPK2-T566 site in HIPK2 does not affect the nuclear speckle localization of the kinase but might contribute to its proliferation-suppressing activity.

**Figure 2:**
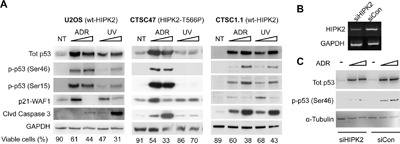
Endogenous HIPK2 (WT *vs*. T566P) response to ADR and UV **A**. WB analysis of the indicated proteins was performed on total cell extracts from wt HIPK2 carrying U2OS and CTSC1.1 cells and HIPK2-T566P carrying CTSC47 cells, prepared 24 hrs after treated with two doses of ADR (0.6 and 3 μM) and UV (50 and 150 J/m^2^). α-Tubulin was used as loading control. Cell viability, determined by Trypan blue exclusion test before cell lysis, is reported as percentage at the bottom of each lane. **B**. To reduce the expression level of the endogenous HIPK2, CTSC47 cells were transfected with HIPK2-specific siRNAs (siHIPK2) while the UNC siRNA was used as control (siCon). The mRNA expression level was measured by semi-quantitative RT-PCR. GAPDH was used as loading reference. **C**. Deplete (siHIPK2) and control (siCon) CTSC47 cells were treated with two doses of ADR (0.6 and 3 μM) for 24 hrs. Total p53 and its phosphorylation at Ser46 were analyzed by WB. α-Tubulin was used as a loading control.

Exogenous HIPK2 overexpression is thought to mirror HIPK2 activity in DDR, while developmental and cytokinesis functions of HIPK2 are better revealed by loss-of-function strategies with rescue experiments in HIPK2-defective cells that can be obtained only with very low expression of the exogenous kinase [32–34]. Thus, based on our overexpression data, we sought to assess the DDR activity of the endogenous HIPK2-T566P mutant. The CTSC47 cells expressing this mutant and carrying wt p53, as assessed by whole-exome sequencing (Supplementary Table 2), were treated with UV and doxorubicin (Adriamycin – ADR), two strong HIPK2 activators whose activity on HIPK2-defective cells is significantly impaired [35]. As demonstrated by p53-S46 phosphorylation, p21-WAF1 expression, caspase 3-cleavage, and reduction of cell viability, U2OS control cells and the CTSC1.1 patient-derived cells expressing HIPK2 and p53 in the wild-type form, did respond to both treatments (Figure 2A, left and right panel) [11, 12]. By contrast, the T566P-expressing CTSC47 did respond to ADR treatment but did not to UV-irradiation (Figure 2A, middle panel), suggesting the existence of an agent-specific impairment.

In response to ADR, DYRK2 was also shown to phosphorylate p53-S46 [36]. Thus, we asked whether the preserved response to ADR by the HIPK2-T566P-carrying CTSC47 cells might be independent of HIPK2 activity. HIPK2-specific siRNAs were employed to deplete HIPK2 expression in the CTSC47 cells as previously described [37] (Figure 2B). These primary cells grow as spheroids and cannot be efficiently transfected. However, the mild downregulation of HIPK2 was associated to a reduction of p53-S46 phosphorylation in response to ADR (Figure 2C) indicating that the HIPK2-T566P mutant does contribute to ADR-induced p53 activation. Together, the results we obtained on the endogenous HIPK2 suggest a differential role for the HIPK2-T566 site in cell response to UV and ADR.

**Figure 3:**
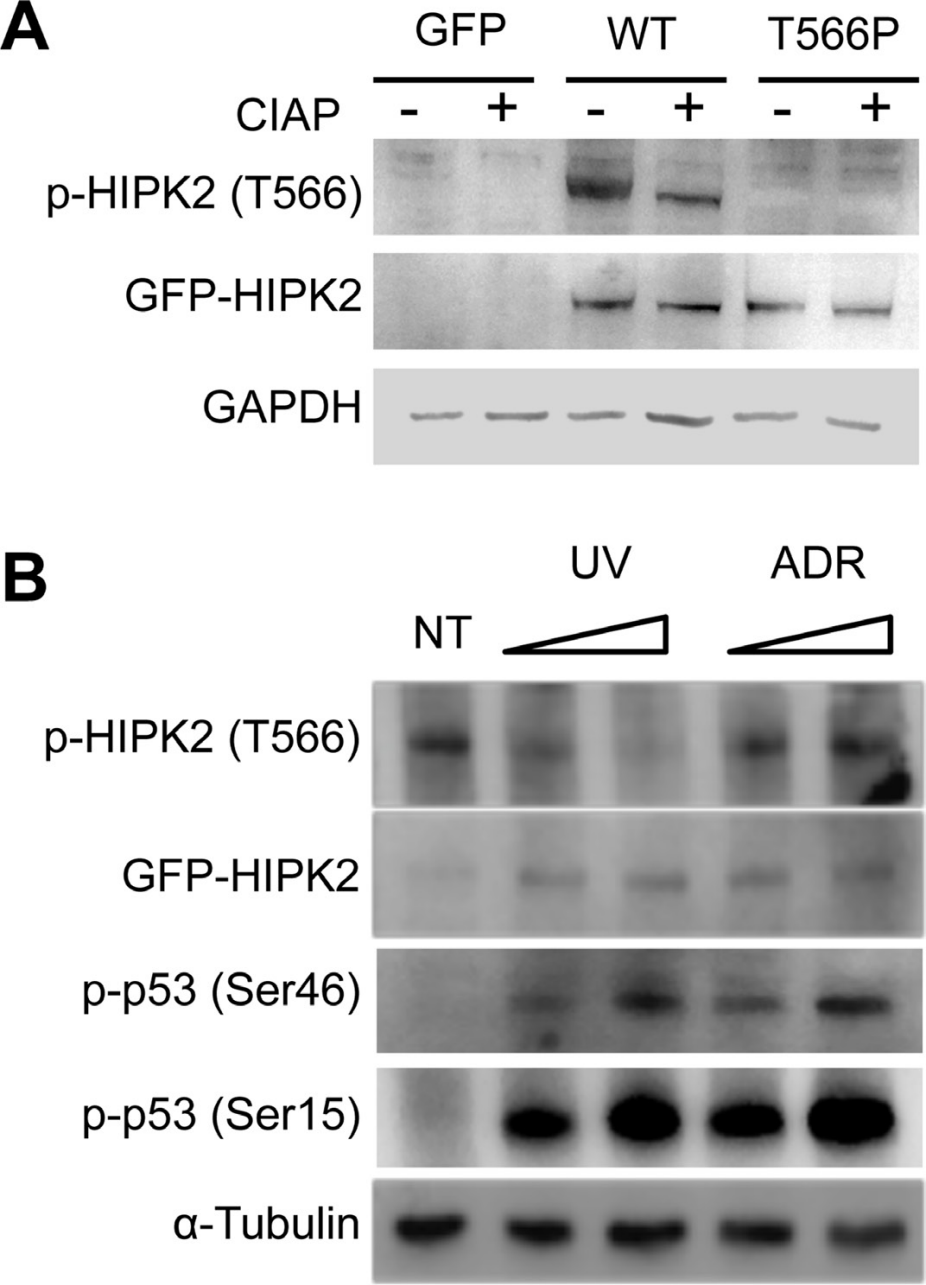
HIPK2-T566 phosphorylation analysis **A**. U2OS cells were transfected with the indicated GFP-tagged vectors and total cell extracts analyzed by WB before and after phosphatase (CIAP) treatment. The anti-p-HIPK2 (T566) Ab recognizes the WT protein but not the non-phosphorylatable HIPK2-T566P mutant and gives a strongly reduced signal in the dephosphorylated WT sample. The amount of GFP-tagged HIPK2 proteins (WT and T566P) was detected by anti-GFP Ab. GAPDH was used as loading control. **B**. U2OS cells were transfected with the GFP-tagged wt HIPK2 and irradiated with UV (50 and 150 J/m^2^) or treated with ADR (0.6 and 3 μM). Non-treated (NT) cells were used as control. Total cell extracts were prepared 24 hrs post-treatments and analyzed by WB for the indicated total and phosphorylated proteins.

Our previous data by liquid chromatography tandem mass spectrometry (LC-MS/MS) on mouse Hipk2 showed that Hipk2-T566 (T559) is an autophosphorylation site and its phosphorylation is lost in the catalytic-impaired K228R (K221R) and Y361F (Y354) mutants [20 and unpublished results]. T566 was not found phosphorylated in the human HIPK2 by the same analyses [19]. Since negative results by LC-MS/MS do not necessarily indicate the absence of the event and our in silico and functional analyses supported the relevance of the T566P substitution, we developed a T566-phospho-specific Ab (see Materials and Methods) to evaluate the contribution of this phospho-site to UV- and ADR-induced DDR. As shown in Figure 3A, the anti-p-HIPK2 (T566) Ab was demonstrated to immunoreact with human wt HIPK2 while it did not recognize the non-phosphorylatable HIPK2-T566P mutant. In addition, treatment with alkaline phosphatase significantly reduced Ab immunoreactivity, further supporting the Ab specificity and confirming the occurrence of phosphorylation at T566 in the kinase. Next, we employed this Ab to assess HIPK2-T566 phosphorylation in UV- and ADR-induced DDR. U2OS cells expressing the GFP-tagged wt HIPK2 were UV-irradiated or treated with ADR. Untreated cells were used as control. As for other HIPK2 S/T sites, we found that HIPK2-T566 was constitutively phosphorylated in the untreated cells (Figure 3B). Interestingly, UV-irradiation was associated with dephosphorylation at T566, while ADR-treatment did not modify the T566 phosphorylation status (Figure 3B), though, as expected for a wt HIPK2, both treatments resulted in a comparable p53 phosphorylation at S46, supporting a differential contribution of the HIPK2-T566 phosphorylation status in the UV- and ADR-induced HIPK2 activation.

**Figure 4:**
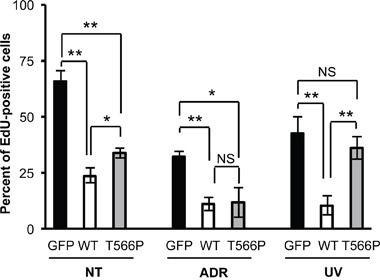
Comparison of the effects induced by UV and ADR treatments on wt HIPK2 and HIPK2-T566P overexpressing cells U2OS cells were transfected and treated as in Figure 3B (ADR, 0.6 μM; UV, 50 J/m^2^) and analyzed after 48 hrs. Inhibition of cell proliferation was assessed by EdU-incorporation and subsequent detection by fluorescence. Data represent the mean ± SE of at least three different experiments. * P<0.05; ** P<0.001; NS P>0.05 by Student t test.

To verify this differential contribution, U2OS cells were transfected with GFP-tagged wt HIPK2 or the T566P mutant; the GFP-empty vector was transfected as negative control. The three transfected populations were treated with ADR, UV-irradiated, or maintained in non-treated conditions. As expected from the above reported data (Figure 1C), EdU incorporation was reduced by exogenous wt HIPK2 expression and, to a lesser extent, by the T566P mutant (Figure 4, NT bars). ADR-treatment resulted in a comparable, further reduction of EdU incorporation in both wt HIPK2 and HIPK2-T566P expressing cells (Figure 4, ADR bars). In contrast, UV-irradiation further reduced EdU incorporation only in the wt HIPK2 expressing cells, while those expressing the T566P mutant behaved as the GFP-control cells (Figure 4, UV bars). These results strongly support the conclusion that the phosphorylation at T566 contributes to UV-mediated reaction of HIPK2 but it is dispensable for the doxorubicin-induced response of the kinase. Taken together, these data show, for the first time, that different genotoxic agents may activate HIPK2 by diversely modulating its autophosphorylation at a single site.

The involvement of HIPK2 in a multitude of biological processes and its participation in many different signaling pathways is made possible by PTMs that increase its functional diversity. In this light, the finding that a specific HIPK2 autophosphorylation site, T566, diversely contributes to UV- and doxorubicin-mediated DDR may not be surprising. However, thus far, HIPK2 activation and function has been indiscriminately studied upon UV, IR or chemotherapeutic drug treatments. For example, HIPK2 autophosphorylation at T880/S882 and Pin1 binding have been studied upon doxorubicin treatment [22]; c-Abl-mediated phosphorylation at Y367 upon UV and IR [23]; AMPKα2-mediated phosphorylation at T119, S121, and T1114 upon IR [24]. While generalizations of these results are usually not specifically claimed, we should be aware that the common definition of HIPK2 activation in DDR implies at least diverse phosphorylation states. This should prompt us to expend more effort in developing tools for studying the endogenous protein in both physiological and pathological conditions.

## MATERIALS AND METHODS

### Whole-exome sequencing and data analysis

Target enrichment was performed using in-solution technology (NimbleGen SeqCap EZ Library v.3.0, Roche), and the resulting target libraries were sequenced by Illumina sequencing technology (HiSeq2000). Raw image files were processed by Illumina basecalling software (CASAVA 1.7) using default parameters. Paired-end reads were aligned to the human genome (UCSC GRCh37/hg19) with the Burrows-Wheeler aligner (BWA v. 0.7.10) [38]. Presumed PCR duplicates were removed using Picard's MarkDuplicates. The Genome Analysis Toolkit (GATK 3.3) [39] was used for realignment of sequences encompassing indels and for base quality recalibration. Somatic single-nucleotide variants were detected using Mutect software v.1.1.6 [40] and small indels were identified through a comparison between indels called in individual C-CSC lines and their matched nontumoral samples by means of the GATK Haplotype Caller algorithm [41], as previously described [42, 43]. The resulting SNVs and small indels were annotated by SnpEff v3.6 [44] and dbNSFP2.8 [45] in terms of functional impact of variants [46, 47]. Variant validation and genotyping were performed by direct sequencing using the ABI BigDye Terminator Sequencing kit (Applied Biosystems) and an ABI3500 capillary sequencer (Applied Biosystems).

### Cells culture and RNA interference

The human osteosarcoma U2OS cells were cultured in DMEM supplemented with 10% (v/v) FBS and antibiotics (Invitrogen). The patient-derived colon spheroids were kindly provided by Prof R. De Maria and cultured in a serum-free medium supplemented with 20 ng/ml EGF and 10 ng/ml FGF-2 (PrePro –Tech) [48]. For DDR, subconfluent cells were irradiated with UV-B using a Vilbert Lourmat Irradiator or incubated in the presence of doxorubicin (Adriamycin; Sigma).

RNA interference was obtained by HIPK2-specific Stealth RNAi sequences and Stealth RNAi-negative control (UNC) (Invitrogen), as previously described [37]. Cells were transfected by RNAi-MAX Lipofectamine (Invitrogen) according to manufacturer's instructions and BLOCK-iT Red Fluorescent oligo (Invitrogen) was used to measure RNA transfection efficiency.

### RT–PCR

Total RNA was isolated using the RNeasy mini Kit (Qiagen). cDNA was synthesized using a M-MLTV RTase and amplified with GoTaq DNA polymerase (Promega). The following primers were employed (NCBI RefSeq NM_022740.4):

*HIPK2*-fw: ggctgaccggcgggagtt; *HIPK2*-rev: ggtcaggccgggcacaaatct.

PCR-amplifications were performed in duplicate on two different RNA preparations.

### HIPK2 expression vectors and cell transfection

The pEGFP-HIPK2-FL vector, encoding for a N-terminal GFP-tagged wild type HIPK2-FL protein [37] was mutagenized by QuickChange II Site-Direct Mutagenesis kit (Agilent) with the following primers:

T566P: cacggtgaaccagagcaaaccccctttcatcac;

K565T: acggtgaaccagagcacaacccctttcatcacg.

The obtained pEGFP-HIPK2-T566P and pEGFP-HIPK2-K565T vectors were validated by direct sequencing and transfected in U2OS cells by electroporation (0.2 kV, 950 μF) using a Gene Pulser (Bio-Rad) or by Lipofectamine (Invitrogen), according to manufacturer's instructions.

### *In vitro* kinase assay

Recombinant GFP-HIPK2, GFP-K565T and GFP-T566P were produced in U2OS cells by transfection with Lipofectamine (Invitrogen). Total cell extracts were prepared 24 hrs post-transfection by incubation for 30 min at 4°C in lysis buffer [50 mM Tris-HCl (pH 7.4), 300 mM NaCl, 1% Nonidet P-40, 5mM EDTA, protease and phosphatase inhibitor (Roche)]. When required, to reduce protein phosphorylation, the total cell extracts were incubated at 30°C for 30 min in the presence of 20 U/ml calf intestinal alkaline phosphatase (Sigma). After centrifugation, GFP-fusion proteins were purified from supernatant by overnight incubation with anti-GFP Ab-sepharose beads (Abcam) at 4°C and used as enzymatic source. Western blot (WB) analysis with a different anti-GFP Ab (mouse monoclonal anti-GFP Ab, Roche) was employed to quantify purified proteins. For kinase assay, recombinant proteins were incubated with recombinant p53, p63, and MBP for 30 min at 30°C in a kinase buffer in the presence of [γ^32^P]-ATP, as described [15]. The phosphorylated substrates were resolved on precast NuPAGE 4-12% gels (Thermo Fisher Scientific) and analyzed by autoradiography.

### Western blotting and antibodies

A rabbit polyclonal Ab recognizing the phosphorylated HIPK2-T566 site, anti-p-HIPK2 (Thr566) Ab was raised against a phospho-peptide representing the human HIPK2 region surrounding T566 and affinity purified with the immunizing phosphorylated peptide after negative absorption with the non-phosphorylated peptide. Peptide design, Ab production and purification were all performed by the custom antibody service of Thermofisher.

Total cell extracts were prepared, separated, transferred onto nitrocellulose membrane, and immunodecorated as described [37]. The following, additional Abs were employed: anti-GFP (mouse monoclonals, clones 7.1 and 13.1 - Roche), anti-p53 (mouse monoclonal: clone DO-1 - Santa Cruz Biotechnology), anti-p-p53(Ser46) (rabbit polyclonal - Santa Cruz Biotechnology), anti-p21 (mouse polyclonal - Santa Cruz Biotechnology), anti-p-p53(Ser15) (rabbit polyclonal - Cell Signaling Technology), anti-cleaved caspase-3 (rabbit polyclonal - Cell Signaling Technology), anti-α-tubulin (mouse monoclonal, clone TU-01 - Immunological Science), anti-GAPDH (mouse monoclonal, clone 6C5 - Santa Cruz Biotechnology). Following incubation for 1 hr at room temperature with secondary antibodies, anti-HRP-conjugated goat-anti-mouse and goat-anti-rabbit (Bio-Rad Laboratories), bound Abs were reviled by ECL-WB Detection System (GE Healthcare) and analyzed by chemiluminescence imaging system (UVITEC Cambridge).

### EdU incorporation and detection

Cells were incubated in the presence of 10 μM EdU labeling solution for 3 hrs, fixed in 2% formaldehyde, permeabilized with 0.25% TritonX 100, blocked with 5% BSA an developed according to manufacturing instructions (Click-iT® EdU imaging Kit, ThermoFisher). Nuclei were counterstained with 1X Hoechst® 33342. At least 500 cells per sample were counted. Fluorescent signals were recorded by Olympus BX53 microscope (Olympus Life Science) and images were captured with a ProgRes MFCOOL camera (Jenoptik) at a magnification of 40X.

### Statistical analyses

For comparison between two independent groups, the Student's t-test was used.

## SUPPLEMENTARY FIGURE AND TABLES


