# Supplementary material for: HIPK2-T566 autophosphorylation diversely contributes to UV- and doxorubicin-induced HIPK2 activation
Source: Oncotarget. 2017 Jan 2;8(10):16744–54. doi: 10.18632/oncotarget.14421 (PMC5369998; doi:10.18632/oncotarget.14421)
Supplement: Supplementary file 1 [file oncotarget-08-16744-s001.pdf]

HIPK2-T566 autophosphorylation diversely contributes to UV- and doxorubicin-induced HIPK2 activation

SUPPLEMENTARY FIGURE AND TABLES

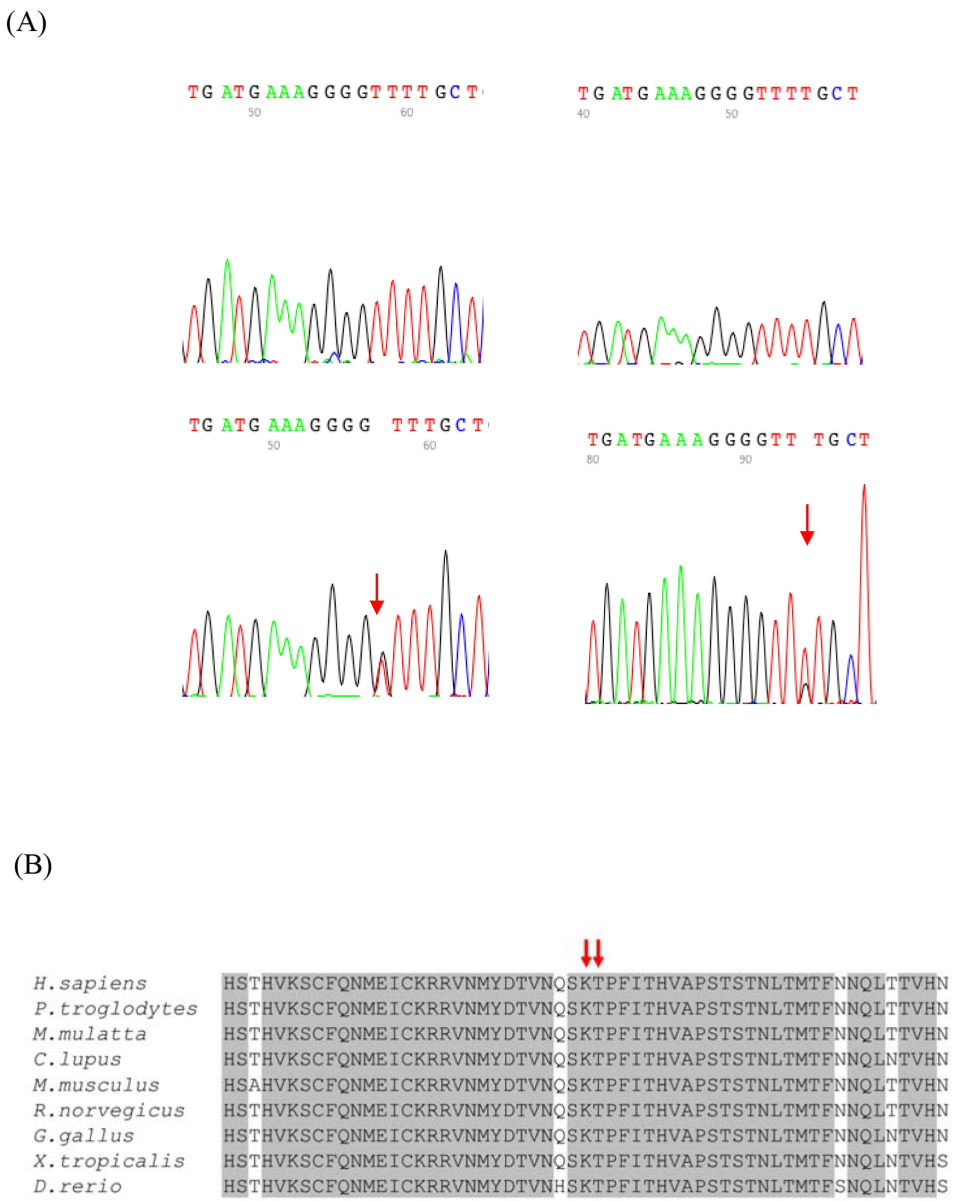

Supplementary Figure 1: Somatically acquired *HIPK2* mutations in C-CSC lines. A. Chromatograms showing the somatic origin of the p.Lys565Thr and p.Thr566Pro in lines CTSC85 and CTSC47, respectively. **B.** Multiple alignments of HIPK2 orthologs showing conservation of Lys565 and Thr566 among vertebrates.

Supplementary Table 1: Data output of WES performed on 24 C-CSC lines

	Median	min	max
**target regions coverage[1] (%)**	99.1	98.0	99.3
target regions coverage > 20X (%)	91.0	86.0	95.5
target regions coverage > 30X (%)	78.5	71.0	93.7
target regions coverage > 50X (%)	45.0	41.0	85.0
median depth of coverage	59	56	112

[1]NimbleGen SeqCap EZ Library v.3.0 (Roche).

Supplementary Table 2: Major driver genes mutation profiling of the CTSC47 C-CSC line

gene	amino acid sequence change
APC	wild type
ATM	p.Ala1812Asp
KRAS	p.Gly12Val
PIK3CA	p.His1047Arg
SMAD4	p.Gln366*<br>p.Arg497His
TP53	wild type
